# Engineering growth phenotypes of *Aspergillus oryzae* for L-malate production

**DOI:** 10.1186/s40643-023-00642-7

**Published:** 2023-04-05

**Authors:** Huiyun Zuo, Lihao Ji, Jingyu Pan, Xiulai Chen, Cong Gao, Jia Liu, Wanqing Wei, Jing Wu, Wei Song, Liming Liu

**Affiliations:** 1 State Key Laboratory of Food Science and Technology, Jiangnan University, 1800 Lihu Road, Wuxi, 214122 Jiangsu China; 2 International Joint Laboratory on Food Safety, Jiangnan University, Wuxi, 214122 China; 3 School of Pharmaceutical Science, Jiangnan University, Wuxi, 214122 Jiangsu China

**Keywords:** L-malate, *Aspergillus oryzae*, Adaptive evolution, Growth mechanism, Growth phenotypes

## Abstract

Improving the growth status of *Aspergillus oryzae* is an efficient way to enhance L-malate production. However, the growth mechanism of filamentous fungi is relatively complex, which limits *A. oryzae* as a cell factory to produce L-malate industrially. This study determined the relationship between growth status and L-malate production. The optimal ranges of colony diameter, percentage of vegetative mycelia, and pellet number of *A. oryzae* were determined to be 26–30 mm, 35–40%, and 220–240/mL, respectively. To achieve this optimum range, adaptive evolution was used to obtain the evolved strain Z07 with 132.54 g/L L-malate and a productivity of 1.1 g/L/h. Finally, a combination of transcriptome analysis and morphological characterization was used to identify the relevant pathway genes that affect the growth mechanism of *A. oryzae*. The strategies used in this study and the growth mechanism provide a good basis for efficient L-malate production by filamentous fungi.

**Graphical Abstract:**

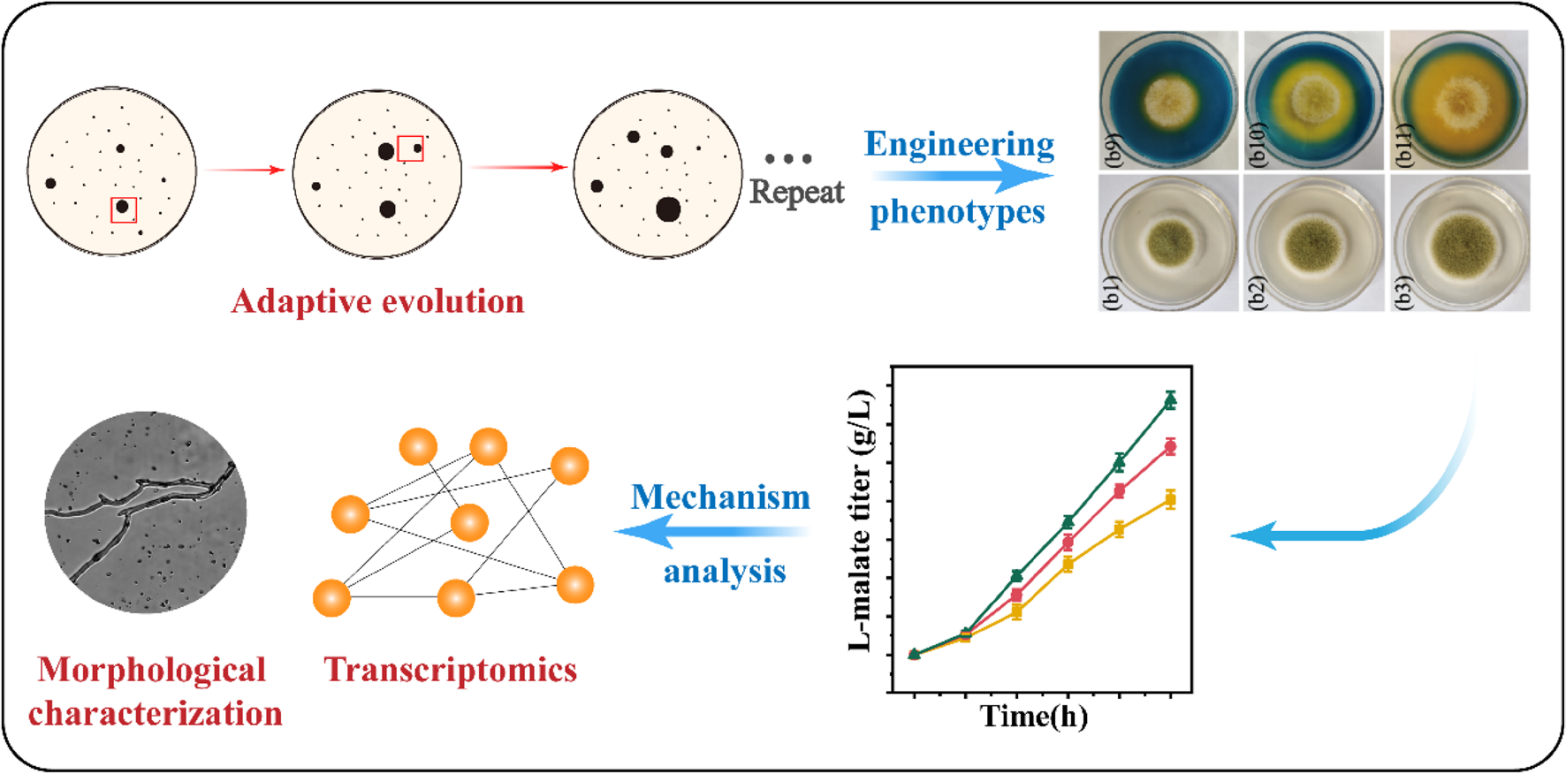

**Supplementary Information:**

The online version contains supplementary material available at 10.1186/s40643-023-00642-7.

## Highlights

- A close correlation was found between the growth status of Aspergillus oryzae and L-malate production.
- Adaptive evolution enhances growth status and capacity of L-malate production of Aspergillus oryzae.
- Transcriptomic analysis and morphological characterization reveal the growth mechanism of Aspergillus oryzae.

**Supplementary Information:**

The online version contains supplementary material available at 10.1186/s40643-023-00642-7.

## Introduction

As an important four-carbon dicarboxylic acid, L-malate is widely used in beverage, food chemical synthesis, and flavor industries (Goldberg et al. 2006) (Ljubimova et al. 2008; Thakker et al. 2015). There are many ways to efficiently produce L-malate, among which microbial fermentation is more renewable and environmentally friendly. In recent decades, various native and metabolically-engineered strains have been used to produce L-malate, including *Aspergillus flavus* (Battat et al. 1991), *Aspergillus niger* (Iyyappan et al. 2018; West 2011; Xu et al. 2020), *Rhizopus delemar* (Ye et al. 2013), *Saccharomyces cerevisiae* (Nakayama et al. 2012; Taing and Taing 2006), *Escherichia coli* (Dong et al. 2017; Guo et al. 2018; Zhang et al. 2011), and *Ustilago trichophora* (Zambanini et al. 2017, 2016). Among the various strains, *A. flavus* was the first natural strain used for the synthesis of L-malate by optimizing the fermentation parameters to achieve an L-malate titer and productivity of 113 g/L and 0.59 g/L/h, respectively. However, because of the presence of carcinogenic aflatoxins, *A. flavus* has never been used for large-scale production (Battat E. 1991). In addition, the plant pathogen *U. trichophora* can produce 196 g/L L-malate after 10–12 days of fermentation (Zambanini et al. 2016). *E. coli* was chosen as the host strain to engineer the production of 34 g/L L-malate with a productivity of 0.47 g/L/h in a two-stage fermentation process (Zhang et al. 2011). Although *E. coli* is an ideal strain for metabolic pathway modification, its L-malate production capacity does not exceed that of filamentous fungi.

*Aspergillus oryzae*, widely used in the food industry, is a promising strain for L-malate production (Brown et al. 2013; Ji et al. 2021). Approaches to improve the L-malate production capacity of *A. oryzae* include metabolic engineering modifications, fermentation process optimization, and random mutations. (i) Metabolic engineering strategies include the overexpression of key enzymes (Liu et al. 2017), knockout of by-product genes (Liu et al. 2018), and enhancement of L-malate transport (Brown et al. 2013; Knuf et al. 2013). For instance, when there is an overexpression of the C4-dicarboxylic acid transporter (C4T318), pyruvate carboxylase (*pyc*), and malate dehydrogenase (*mdh*) in *A. oryzae*, the titer and productivity of L-malate increased to 154 g/L and 0.94 g/L/h, respectively, in 164 h (Brown et al. 2013). (ii) Fermentation process parameters, such as the carbon source (Dörsam et al. 2017), nitrogen source (Ding et al. 2018; Knuf et al. 2013; Ochsenreither et al. 2014), and agitation speed (Chen et al. 2019), can affect the production of L-malate. For example, a novel nitrogen supply strategy (the initial tryptone concentration was 6.5 g/L and was supplemented with 3 g/L tryptone at 24 h) was developed to increase the titer of L-malate from 130.5 g/L to 164.9 g/L in a 30-L fermenter (Ji et al. 2021). (iii) To get a strain with excellent phenotype, a series of random mutagenesis strategies, including chemical mutagens (Ding et al. 2018; Ji et al. 2021) and physical mutagens (Chen et al. 2019), such as ^60^Co-γ irradiation, atmospheric pressure room temperature plasma (ARTP), were used solely or in combination. For example, Chen et al. successfully screened *A. oryzae* FCD15 that could produce 52.9 g/L L-malate at a productivity rate of 0.40 g/L/h using ^60^Co-γ irradiation and ARTP mutagenesis (Chen et al. 2019).

However, although different methods have been employed and much progress has been made in L-malate production by *A. oryzae*, L-malate is not so enough to large-scale production. This is because the growth mechanism of filamentous fungi is more complicated, and the relatively poor growth status limits industrial application (Iyyappan et al. 2018; Pringle and Taylor 2002; Schoustra and Punzalan 2012). Therefore, clarifying the growth mechanism and controlling the growth status of *A. oryzae* is key to enhance the industrial production of L-malate.

In this study, the relationship between the growth status and the L-malate production by *A. oryzae* was built and then the optimum range of growth parameters was obtained. Based on this optimal range, adaptive evolution was used to improve the growth status of *A. oryzae*, and the evolved strain Z07 which synthesis 132.5 g/L L-malate in a 7.5-L fermenter, was obtained. Furthermore, a combination of transcriptomic analysis and morphological characterization was used to investigate the growth mechanisms of *A. oryzae*.

## Materials and methods

### Strain and media

The parental strain *A. oryzae* Z01 used in this paper was conserved at the China Center for Type Culture Collection (CCTCC) under the preservation number of CCTCC NO: M 2016401. The seed medium was composed of 60 g/L glucose, 3 g/L tryptone, 750 mg/L KH_2_PO_4_, 980 mg/L K_2_HPO_4_·3H_2_O, 100 mg/L MgSO_4_·7H_2_O, 75 mg/L CaCl_2_, and 1 mL/L 1000 × micronutrient solution (5 g NaCl, 0.15 g FeSO_4_·7H_2_O, and 1 L water). The acid-production medium was composed of 120 g/L glucose, 80 g/L CaCO_3_, 6 g/L tryptone, 200 mg/L KH_2_PO_4_, 200 mg/L K_2_HPO_4_·3H_2_O, 100 mg/L MgSO_4_·7H_2_O, 75 mg/L CaCl_2_, and 1 mL/L 1000 × micronutrient solution. When we used this medium in 250 mL shake flask, we needed to feed glucose 30 g/L and CaCO_3_ 30 g/L every 48 h. Acid production medium in 7.5-L fermenter was composed of 130 g/L glucose, 30 g/L CaCO_3_, 6 g/L tryptone, 600 mg/L KH_2_PO_4_, 600 mg/L K_2_HPO_4_·3H_2_O, 100 mg/L MgSO_4_·7H_2_O, 75 mg/L CaCl_2_, 8 mg/L MnSO_4_, and 1 mL/L × 1000 micronutrient solution.

### Culture conditions

Spore suspensions were obtained by incubating the strains at 35 °C for 3 days and then using a 0.05% Tween solution to collect the mature spores. The spore suspension was inoculated into a 500 mL flask containing 150 mL of seed medium at a concentration of 1.5 × 10^8^ spores/mL, and the temperature and incubation time for seed fermentation were 34 °C and 24 h. We then inoculated the cultured seeds into a sterile 250 mL flask containing 50 mL of fermentation medium at 10% inoculum, and the incubation conditions were 36 °C. Next, the seeds will be inoculated into 250 mL sterile flasks containing 50 mL of fermentation medium at 10% inoculum level and incubated at 36 °C, 200 rpm/min and 120 h, with the addition of 30 g/L CaCO_3_ and 30 g/L glucose during this period. The fermentation was scaled up to a 7.5-L fermenter (INFORS infors, Switzerland) with the same inoculum concentration as the shake flask, but with a two-stage temperature controlling strategy to control the fermentation temperature (36 °C for the first 18 h and 32 °C until 120 h) and a agitation status of 600 rpm/min. 40 g/L CaCO_3_ was added every 24 h for the first 72 h to maintain the pH of the fermentation broth at 6.0 or higher, while glucose was added to the fermenter at a constant flow rate to maintain the glucose concentration above 25 g/L. The same culture conditions were used for all *A. oryzae* mutants.

### ARTP and LiCl mutagenesis

Spore was cultured in PDA medium for 4 days and was washed with aseptic water, and gradient dilution to 10^6^ individual /mL was performed. For atmospheric and room temperature plasma (ARTP) mutagenesis system, 10-μL diluted culture liquid was transferred to the middle of the sterile metal slide. Power and gas of ARTP mutagenesis system are fixed at 100 W and 10 SLW; 10 μL of the spore suspension was exposed to ARTP jet for different treatment times ranging from 90 to 180 s, which resulted in 70–80% lethality rate and then spore suspension was diluted to 10^2^ and 10^3^ individual /mL for coating and screening.

The culture method of spore is the same as of the ARTP. For LiCl mutagenesis, the suspended spores were treated with 0.5 mg/mL to cause mutagenesis, and cultured in an oscillator at 34 °C for 30 min. Next, spore suspension was diluted to 10^2^ and 10^3^ individual/mL for coating and screening.

### Adaptive evolution experiment

During adaptive evolution, 1.6 μL/mL to 2.0 μL/mL 2-phenylethanol was added to potato glucose medium and approximately 3.5 × 10^4^ spores were inoculated into the medium at 35 ℃ for approximately 4 days. When the incubation was completed, the areas of more vigorous mycelial spore cultivation were collected with Tween solution and were again inoculated into fresh medium. This step was repeated continuously until the growth of the strain was comparable to the growth of the control (without 2-phenylethanol). Then purified the evolved strains and the colony that grew with the strongest growth on PDA plates containing 2-phenylethanol was selected for further stability analysis as well as studies.

### Morphology characterization

The morphology of colonies (such as colony diameter, PVM values, densities, etc.) was determined by the camera (Nikon D70, Nikon Corporation, Japan). The morphological characteristics of mycelium, spores, and pellets were determined by image analysis using scanning electron microscopy (SEM, FEI). Making internal sections of mycelial pellets and then observing them with transmission electron microscopy (TEM, FEI). The number and morphology of mycelial pellets in 7.5L fermenters were determined using an iBrightTM imaging system (Thermo Fisher Scientific Company, America). Spores were incubated in PDA medium for 4 days and mycelial pellets were cultured in acid-producing medium for 7 days, which needed to be diluted with sterile water to avoid overlap affecting observation when morphological observations were made. All spores, mycelial branches and mycelial pellets were pictured under the same conditions.

### Transcriptome sequencing analysis

Spores of evolved strains were cultured in fermentation medium, cells were collected, washed twice with phosphate-buffered saline, and centrifuged at 3500 g for 10 min at 4 °C. Total RNA was isolated using the MiniBEST universal RNA extraction Kit (TaKaRa Bio, Shiga, Japan). Total RNA concentration and mass were determined by microspectrophotometry using an Agilent 2100 bioanalyzer (Agilent Technologies, Santa Clara, CA) for determination. The collected strains were frozen at − 80 °C and sent to the Majorbio Institute for global gene analysis.

### Analytical methods

In order to dissolve excess CaCO_3_ and precipitated acids, whole broth samples for dry cell weight analysis were diluted with 2 M HCl. Biomass was recovered from acidified broth by centrifugation, washed with water and recentrifuged, and dried at 60 °C for a minimum of 48 h before weighing. Quantitation of C4 acids and glucose were performed by reverse-phase high-performance liquid chromatography (HPLC) using an Aminex^®^ HPX-87H ion (300 mm × 250 mm) exclusion column eluted with a refractive index detector and UV detector at 210 nm. The mobile phase was a 5-mM H_2_SO_4_ solution at a flow rate of 0.6 mL/min and a column temperature of 35 °C. The injection volume was 10 μL (Ding et al. 2018).

## Results

### Effect of *A. oryzae* growth status on L-malate production

**Fig. 1:**
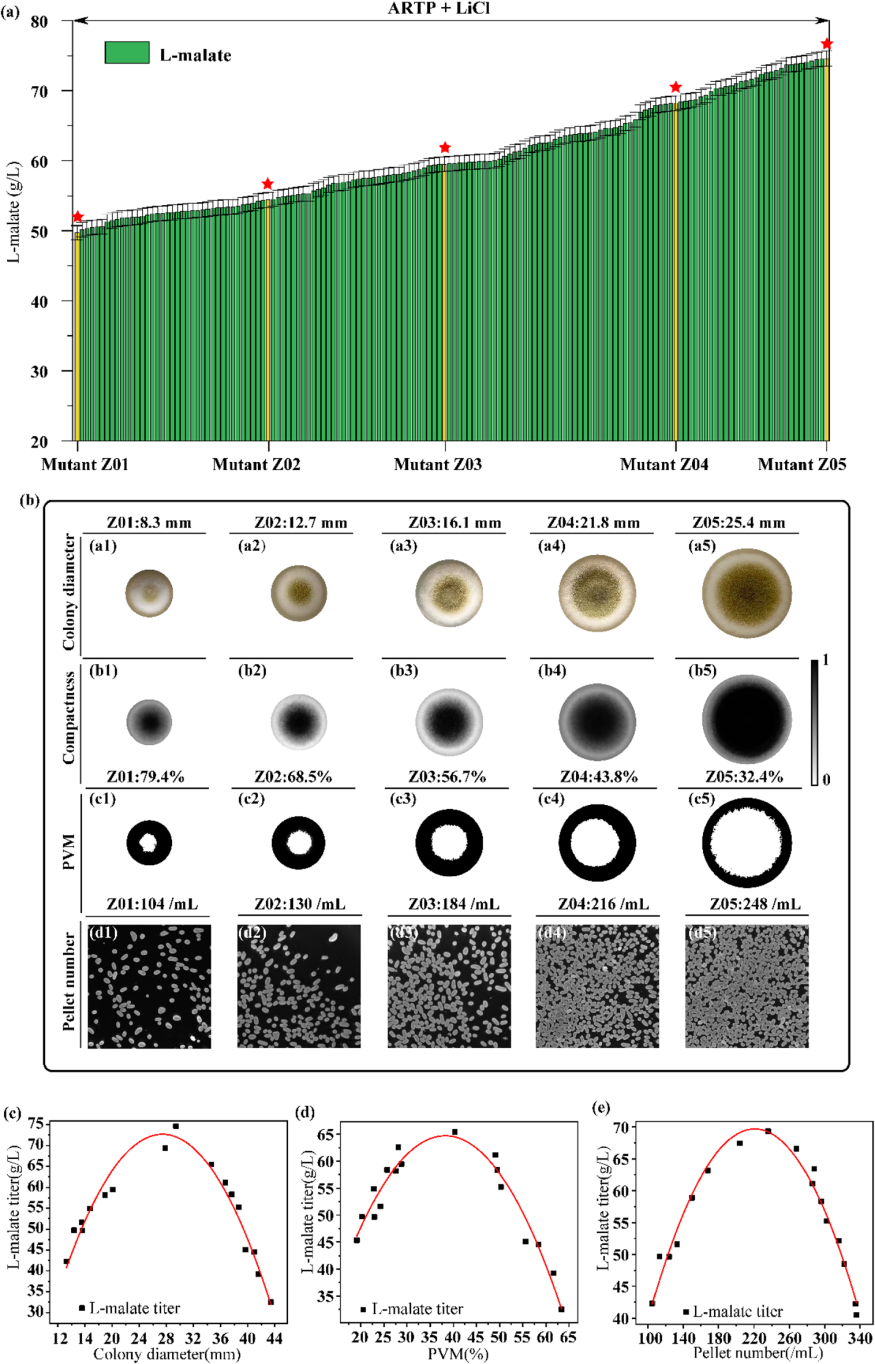
Effect of the *A. oryzae* growth status on L-malate production. **a** The mutant library was constructed by ARTP irradiation and LiCl mutagenesis. **b** Images of the colonies (a1–a5), compactness (b1–b5), percentage of vegetative mycelia (PVM) of colonies(c1–c5) and pellet number (d1–d5) of the five mutants. **c** The relationship between the colony diameter and L-malate titer meet $Y = \, - 0.{15}X^2 + {7}.0{6}X - {1}0.{72 }\left( {R^{2} = 0.{95}} \right)$. **d** The relationship between PVM and L-malate titer meet $Y = \, - 0.0{5}X^2 + {4}.{92}X - {53}.{89 }\left( {R^{2} = 0.{94}} \right)$. **e** The relationship between pellet number and L-malate titer meet $Y = \, - 0.00{2}X^2 + 0.{82}X - {12}.{94 }\left( {R^{2} = 0.{97}} \right)$

To study the effect of *A. oryzae* growth status on L-malate production, a mutant library (150 mutants) was first constructed via the compound mutagenesis of ARTP and LiCl (Fig. 1a). The L-malate titer, yield, and productivity of the mutant Z05 were 74.6 g/L, 0.74 g/g, and 0.62 g/L/h, respectively, which were 50.1%, 13.85%, and 51.22% higher than the corresponding values of the parent Z01. Among them, five better mutants Z01–Z05 with the L-malate titer ranging from 49.7 g/L to 74.6 g/L (Table 1) were selected to relate to the growth status of *A. oryzae*, including colony diameter, percentage of vegetative mycelia (PVM), and pellet number. When the colony diameter, PVM value, and pellet number of the mutants Z01–Z05 changed to 8.3–25.4 mm, 32.4–79.4%, and 104–248/mL, the L-malate titer, L-malate yield, L-malate productivity, and glucose consumption also changed to 49.7–74.6 g/L, 0.65–0.74 g/g, 0.41–0.62 g/L/h, and 75.9–101.2 g/L, respectively (Fig. 1b). These experimental data showed that (1) the colony diameter (*R*^2^ = 0.95), PVM value (*R*^2^ = 0.94), and pellet number (*R*^2^ = 0.97) were closely correlated with the L-malate titer. (2) The L-malate titer increased with increasing colony diameter (below 26–30 mm) and pellet number (below 220–240/mL) but decreased with increasing PVM value when the PVM value was above 35–40% (Fig. 1c–-e). (3) To increase the L-malate titer, we determined the optimal range for colony diameter, PVM and pellet number were 26–30 mm, 35–40%, and 220–240/ml, respectively.

**Table 1:** Comparisons of fermentation performance of *A. oryzae* wild type and mutants

Parameter	Mutants				
	Z01	Z02	Z03	Z04	Z05
Culture time (h)	120	120	120	120	120
Glucose consumption (g/L)	75.9 ± 2.3	82.1 ± 1.3	88.3 ± 2.0	95.6 ± 2.0	101.2 ± 1.5
Dry cell weight (g/L)	11.47 ± 0.43	12.32 ± 0.23	13.54 ± 0.29	15.28 ± 0.29	16.4 ± 0.34
L-malate titer (g/L)	49.7 ± 1.3	54.4 ± 1.4	59.5 ± 0.8	68.2 ± 0.8	74.6 ± 0.7
Yield of malate on glucose (g/g)	0.65 ± 0.01	0.66 ± 0.02	0.67 ± 0.01	0.71 ± 0.01	0.74 ± 0.02
L-malate productivity (g/L/h)	0.41 ± 0.01	0.45 ± 0.01	0.50 ± 0.01	0.57 ± 0.01	0.62 ± 0.01
Yield of L-malate on DCW (g/g)	4.36 ± 0.08	4.40 ± 0.10	4.40 ± 0.10	4.48 ± 0.10	4.54 ± 0.13

The numbers stated are means of three individual experiments ± standard errors

### Improvement of *A. oryzae* growth status through adaptive evolution

**Fig. 2:**
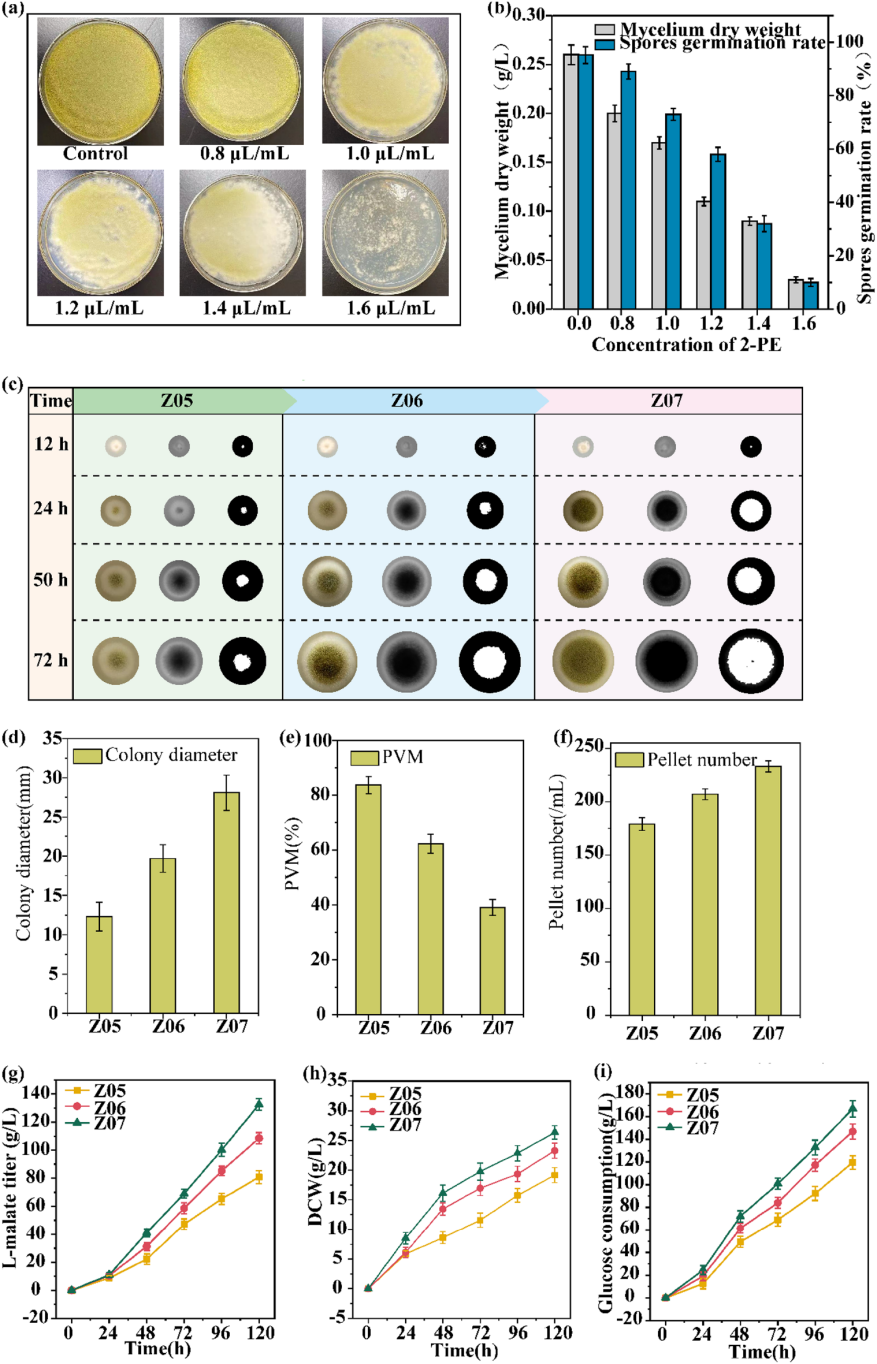
Assessment of the adaptive evolution of *Aspergillus oryzae* strains. **a** Inhibition of *Aspergillus oryzae* by different concentrations of 2-PE.** b** Influence of different concentrations of 2-PE on changes in mycelial dry weight and spores germination rate. **c** Images of colony morphology, compactness and PVM values during the evolution of strain Z05, Z06 and Z07. **d-f** Growth parameters: colony diameter, PVM value and pellet number of strain Z05, Z06 and Z07. **g-i** Fermentation performance parameters: L-malate titer, DCW and glucose consumption of strain Z05, Z06 and Z07 in the 7.5-L fermenter. All data are presented as mean values from three independent experiments. Error bars indicate the standard deviations

An adaptive evolution strategy using 2-phenylethanol (2-PE), a fungal inhibitor, as a stressor was developed to achieve the optimal range of colony diameter (26–30 mm), PVM value (35–40%), and pellet number (220–240/mL) in the strain Z05. 2-PE has an inhibitory effect on the growth of most fungi(Stark et al. 2003), and long-term incubation of *A. oryzae* in an environment containing 2-PE can result in an improved growth status of the strain. When the strain Z05 was cultured in a medium containing 1.6 μL/mL 2-PE, it was almost impossible to produce mature green spores (Fig. 2a). The mycelial dry weight and spore germination rate were only 0.03 g/L and 10.11%, which were 88.46% and 89.38% lower than those of the control (Fig. 2b). Thus, 1.6 μL/mL was chosen as the stressful concentration of 2-PE for achieving adaptive evolution. Through an adaptive evolution strategy involving increasing the concentration of 2-PE from 1.6 μL/mL to 2.0 μL/mL, the 12th generation evolved strain (Z06) and the 25th generation evolved strain (Z07) with enhanced growth status were obtained (Fig. 2c). (i) The colony diameter, PVM values, and pellet number of the strain Z06 were 19.7 mm, 62.3%, and 207/mL, respectively, which were 60.16%, 25.57%, and 15.64% higher than those of the strain Z05; however, the values did not reach the optimal range. (ii) The colony diameter, PVM values, and pellet number of the strain Z07 were 28.1 mm, 39.1%, and 233/mL, respectively, which were 128.46%, 53.29%, and 30.17% higher than those of the strain Z05; these values reached the optimal range (Fig. 2d-f).

The time-curve of the strains Z05, Z06, and Z07 in a 7.5-L fermenter are depicted in Fig. 2g-i and Table 2. (i) The dry cell weight (DCW), L-malate titer, L-malate yield, L-malate productivity, and glucose consumption of the strain Z06 were 21.54%, 34.28%, 8.82%, 34.33%, and 22.83% higher than the corresponding values of the strain Z05. (ii) The DCW, L-malate titer, L-malate yield, L-malate productivity, and glucose consumption of the strain Z07 were 37.45%, 63.99%, 16.18%, 64.18%, and 39.55% higher than the corresponding values of strain Z05. These results indicated that the growth status of *A. oryzae* could enhance the production of L-malate.

**Table 2:** Comparisons of fermentation performance of different strains

Parameter	Strains			Change rate (%)	
	Z05(A)	Z06(B)	Z07(C)	(B-A)/A × 100%	(C-A)/A × 100%
Culture time (h)	120	120	120	–	–
Glucose consumption (g/L)	119.6 ± 1.5	146.9 ± 1.5	166.9 ± 2.2	22.83	39.55
Dry cell weight (g/L)	19.17 ± 0.50	23.30 ± 0.51	26.35 ± 1.01	21.54	37.45
L-malate titer (g/L)	80.8 ± 1.0	108.5 ± 1.7	132.5 ± 2.6	34.28	63.99
Yield of malate on glucose (g/g)	0.68 ± 0.01	0.74 ± 0.01	0.79 ± 0.01	8.82	16.18
L-malate productivity (g/L/h)	0.67 ± 0.01	0.90 ± 0.01	1.10 ± 0.02	34.33	64.18
Yield of L-malate on DCW (g/g)	4.22 ± 0.16	4.66 ± 0.04	5.03 ± 0.12	10.43	19.19

The numbers stated are means of three individual experiments ± standard errors

### Analysis and validation of the genes related to hyphal branching formation

**Fig. 3:**
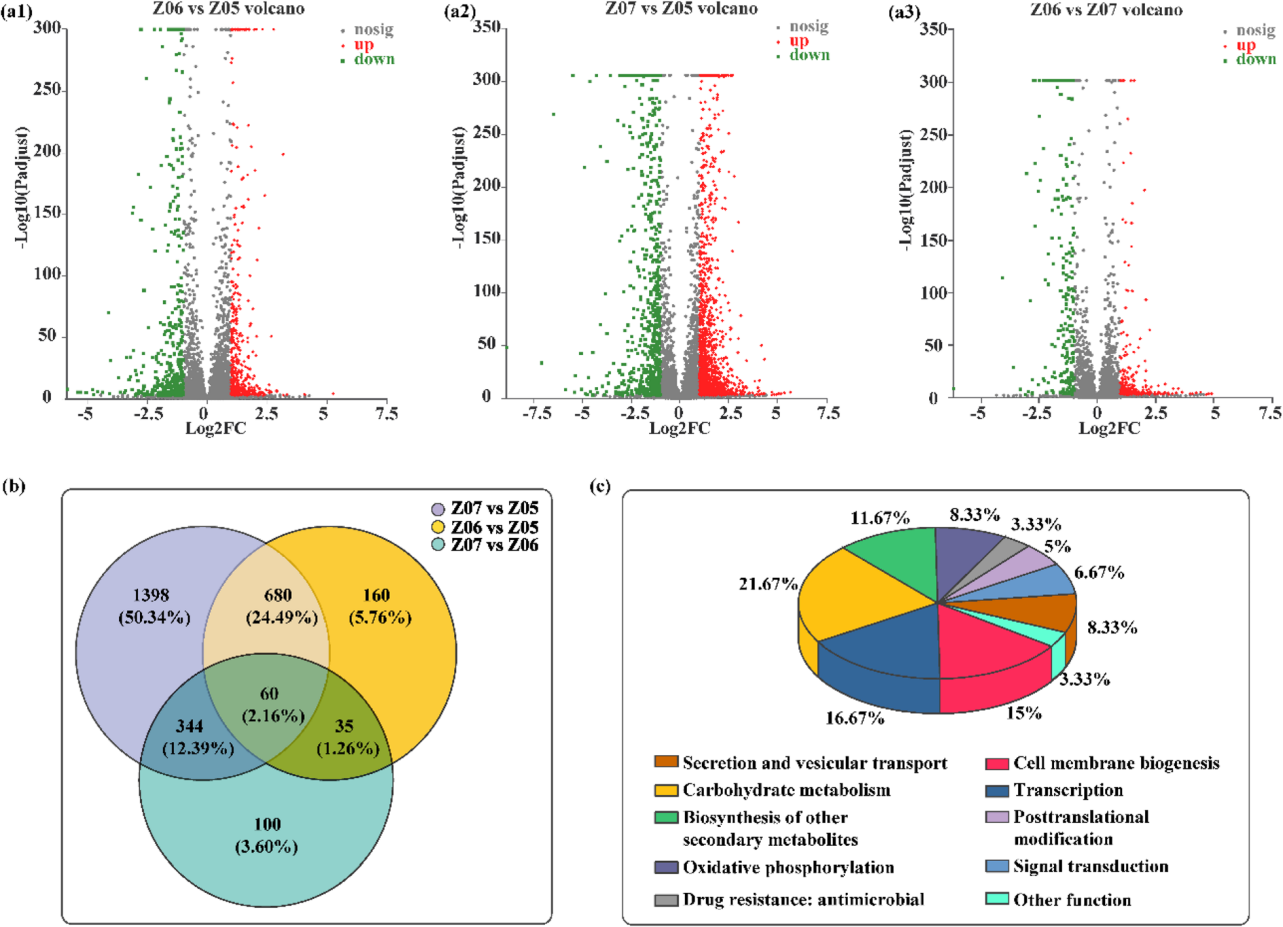
Transcriptome analysis of strain Z05, Z06 and Z07. **a1–a3** Differently expressed genes in strain Z05, Z06 and Z07.** b** Venn diagrams depicting the numbers of commonly regulated genes in strain Z05, Z06 and Z07.** c** Statistical analysis of the metabolic pathways in which the identified significant differentially expressed genes in strain Z05, Z06 and Z07 are involved

The transcriptome data of the strains Z05, Z06, and Z07 were compared to identify the genes that contributed to the change in growth status. The expression levels of 935 genes in strain Z06 were significantly different compared with those in strain Z05 (≥ 2.0-fold change; *P* ≤ 0.05): 485 genes were upregulated, and 450 genes were downregulated (Fig. 3a1). The expression levels of 2482 genes were significantly altered in strain Z07 compared with those in strain Z05: 1651 and 831 genes were upregulated and downregulated, respectively (Fig. 3a2). By comparing the gene expression levels of strains Z06 and Z07, the data revealed 539 genes whose expression changed significantly, including 289 upregulated and 250 downregulated genes (Fig. 3a3). The expression levels of 60 genes were altered in all strains (Fig. 3b). Further, Kyoto Encyclopedia of Genes and Genomes (KEGG) analysis indicated that carbohydrate metabolism, transcription, cell membrane biogenesis, and biosynthesis of other secondary metabolites were the four most notable differentially-regulated pathways, accounting for 21.67%, 16.67%, 15%, and 11.67%, respectively. Oxidative phosphorylation, drug resistance: antimicrobial, posttranslational modification, signal transduction, and secretion and vesicular transport were also affected (Fig. 3c).

**Fig. 4:**
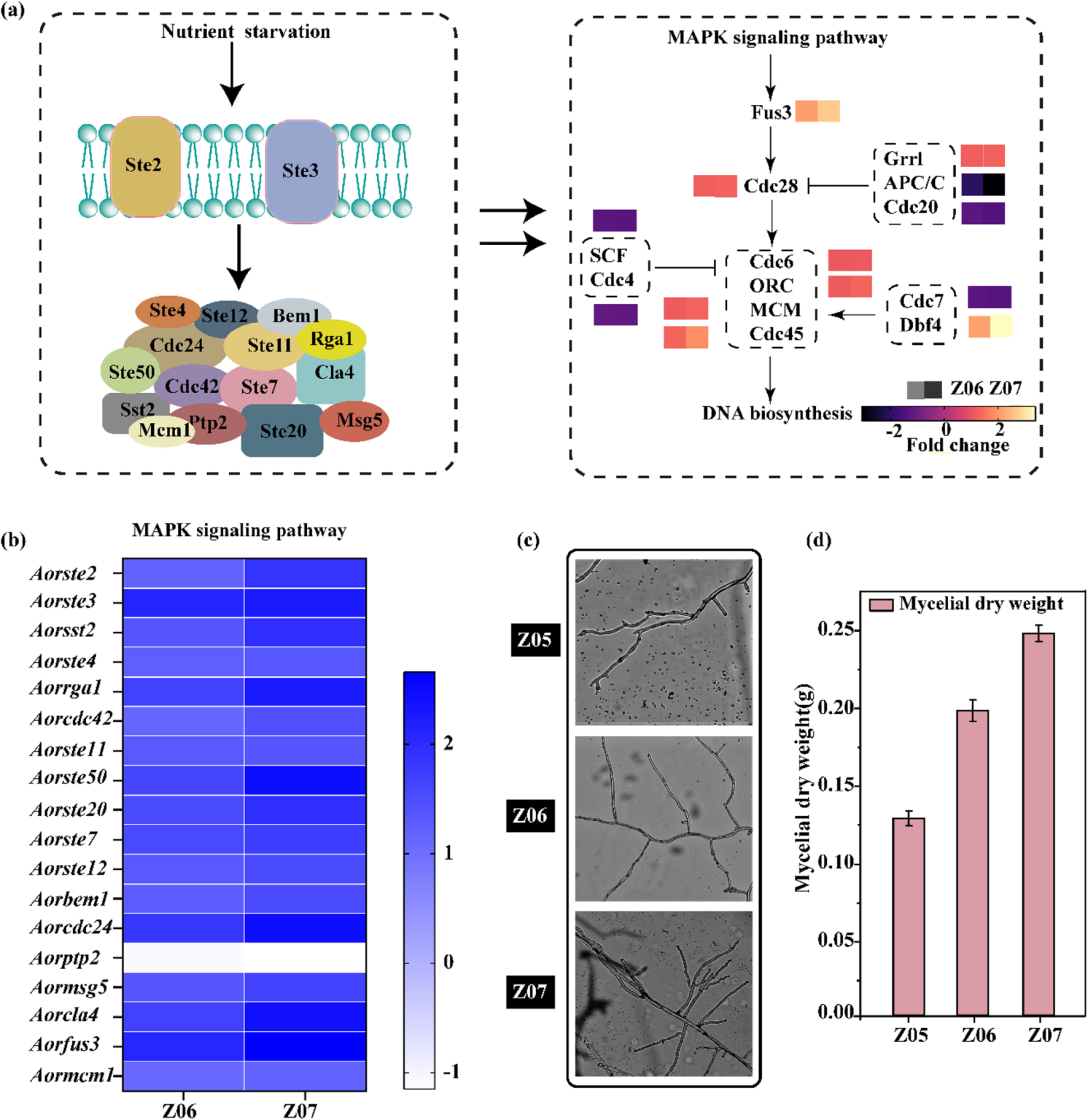
Transcriptome analysis and morphological characterization to clarify the mechanism of hyphal branching formation in *Aspergillus oryzae*.** a** Diagram of the mechanism of hyphal branching formation. **b** Heat map of the hyphal branching formation mechanism following comparison between the strain Z06 and Z07. **c** The morphological characteristics of hyphal branching of the strain Z05, Z06 and Z07 under light microscopy. **d** Mycelial dry weight of the strain Z05, Z06 and Z07. All data are presented as mean values from three independent experiments. Error bars indicate the standard deviations

The transcriptome profiles were compared to elucidate the genes that respond to hyphal branching formation in *A. oryzae* (Fig. 4a). We found that: (i) the expression of genes associated with the MAPK signaling pathway (Fig. 4b), such as *Aorste50* (Z06: 1.61-fold, Z07: 2.44-fold), *Aorste20* (Z06: 1.54-fold, Z07: 1.96-fold), *Aorste7* (Z06: 1.56-fold, Z07: 1.72-fold), *Aorcdc24* (Z06: 1.84-fold, Z07: 2.45-fold), *Aormsg5* (Z06: 1.38-fold, Z07: 1.67-fold), and *Aorptp2* (Z06: − 1.07-fold, Z07: − 1.15-fold), were significantly changed. Among them, the expression level of *Aorcdc24*, a key gene in the MAPK signaling pathway, in strains Z06 and Z07 were 1.84-fold and 2.45-fold higher than that of strain Z05, respectively. (ii) The expression levels of genes with repressive effect on DNA synthesis, such as *Aorapc* (Z06: − 2.02-fold, Z07: − 3.08-fold), *Aorcdc20* (Z06: − 1.28-fold, Z07: − 1.44-fold), *Aorscf* (Z06: − 1.30-fold, Z07: − 1.34-fold), *Aorcdc4* (Z06: − 1.18-fold, Z07: − 1.21-fold), and *Aorcdc7* (Z06: − 1.31-fold, Z07: − 1.4-fold), were also significantly altered. Among them, the expression levels of *Aorapc* in strains Z06 and Z07 were 2.02-fold and 3.08-fold lower than in strain Z05, respectively. Conversely, the expression levels of key genes involved in DNA synthesis, such as *Aorfus3* (Z06: 2.08-fold, Z07: 2.66-fold), *Aororc* (Z06: 1.14-fold, Z07: 1.28-fold), *Aorcdc45* (Z06: 1.29-fold, Z07: 1.88-fold), *Aorcdc28* (Z06: 1.19-fold, Z07: 1.24-fold), *Aodbf4* (Z06: 2.11-fold, Z07: 3.39-fold), and *Aormcm* (Z06: 1.12-fold, Z07: 1.29-fold), were significantly increased. Among them, the expression level of *Aordbf4* in strains Z06 and Z07 were 2.11- and 3.39-folds higher than that of strain Z05, respectively. These data suggest that the MAPK and DNA synthesis pathways were more active in the evolved strains.

The formation of mycelial branching was observed using light microscopy, and the mycelial branching dry weights of these strains were measured. The mycelial branches of strains Z06 and Z07 were denser than those of strain Z05, and the mycelial branches of strain Z07 were the densest (Fig. 4c, d). Similarly, the maximum dry weight of mycelial branches was in strain Z07 with 0.24 g/L, which was 37.0% and 89.6% higher than that of strains Z05 and Z06, respectively. Hyphal branching of *A. oryzae* resulted in a better status during adaptive evolution.

### Analysis and validation of the genes related to conidiophore formation

**Fig. 5:**
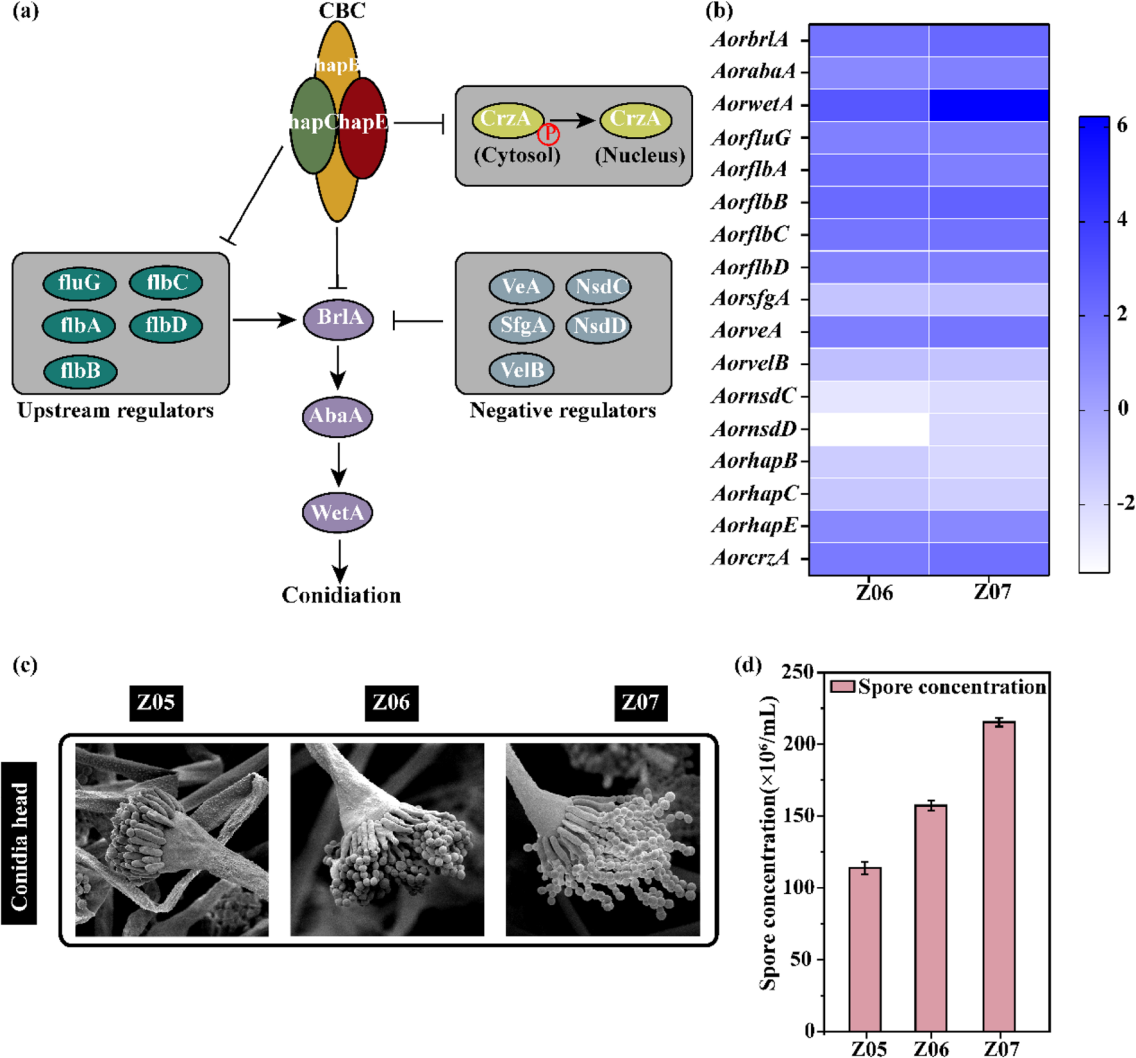
Transcriptome analysis and morphological characterization to clarify the mechanism of conidiophore formation in *Aspergillus oryzae*.** a** Diagram of the mechanism of conidiophore formation. **b** Heat map of the conidiophore formation mechanism following comparison between the strain Z06 and Z07. **c** The morphological characteristics of conidiophore of the strain Z05, Z06 and Z07 under scanning electron microscope. **d** Spore concentration of the strain Z05, Z06 and Z07. All data are presented as mean values from three independent experiments. Error bars indicate the standard deviations

To elucidate the genes that respond to conidiophore formation, transcriptome profiles were compared (Fig. 5a**)**. All the key central conidiation regulators, such as *AorbrlA* (Z06: 1.82-fold, Z07: 2.26-fold), *AorabaA* (Z06: 1.02-fold, Z07: 1.33-fold), and *AorwetA* (Z06: 2.91-fold, Z07: 6.22-fold), and their upstream genes, such as *AorfluG* (Z06: 1.36-fold, Z07: 1.47-fold), *AorfluA* (Z06: 1.39-fold, Z07: 1.96-fold), *AorfluB* (Z06: 2.18-fold, Z07: 2.48-fold), *AorfluC* (Z06: 1.78-fold, Z07: 1.89-fold), and *AorfluD* (Z06: 1.26-fold, Z07: 1.35-fold) were remarkably upregulated. Among them, the expression level of *AorwetA* and *AorflbB* in strains Z06 and Z07 was 2.91-fold and 6.22-fold, 2.18-fold, and 2.48-fold higher than that of the strain Z05, respectively. Conversely, the expression levels of genes, such as *AorsfgA* (Z06: − 1.04-fold, Z07: − 1.23-fold), *AorvelB* (Z06: − 1.04-fold, Z07: − 1.16-fold), *AornsdC* (Z06: − 2.1-fold, Z07: − 2.48-fold), *AornsdD* (Z06: − 1.04-fold, Z07: − 1.89-fold), *AorhapB* (Z06: − 1.5-fold, Z07: − 1.92-fold), and *AorhapC* (Z06: − 1.33-fold, Z07: − 1.65-fold), with inhibitory effects on conidiophore formation were significantly downregulated. Among them, the expression level of the negative regulator *AornsdC* in strains Z06 and Z07 was downregulated by 2.1-fold and 2.48-fold, respectively, compared with that in strain Z05 (Fig. 5b). The morphological characteristics of the conidiophores were observed using scanning electron microscopy (SEM). The conidial heads of strains Z06 and Z07 were more mature than those of strain Z05, and the spores of strain Z07 were the densest (Fig. 5c). The spore concentration of strain Z06 and Z07 was 1.55 and 1.94 times higher than that of strain Z05, respectively (Fig. 5d).

### Analysis and validation of the genes related to mycelial pellet formation

**Fig. 6:**
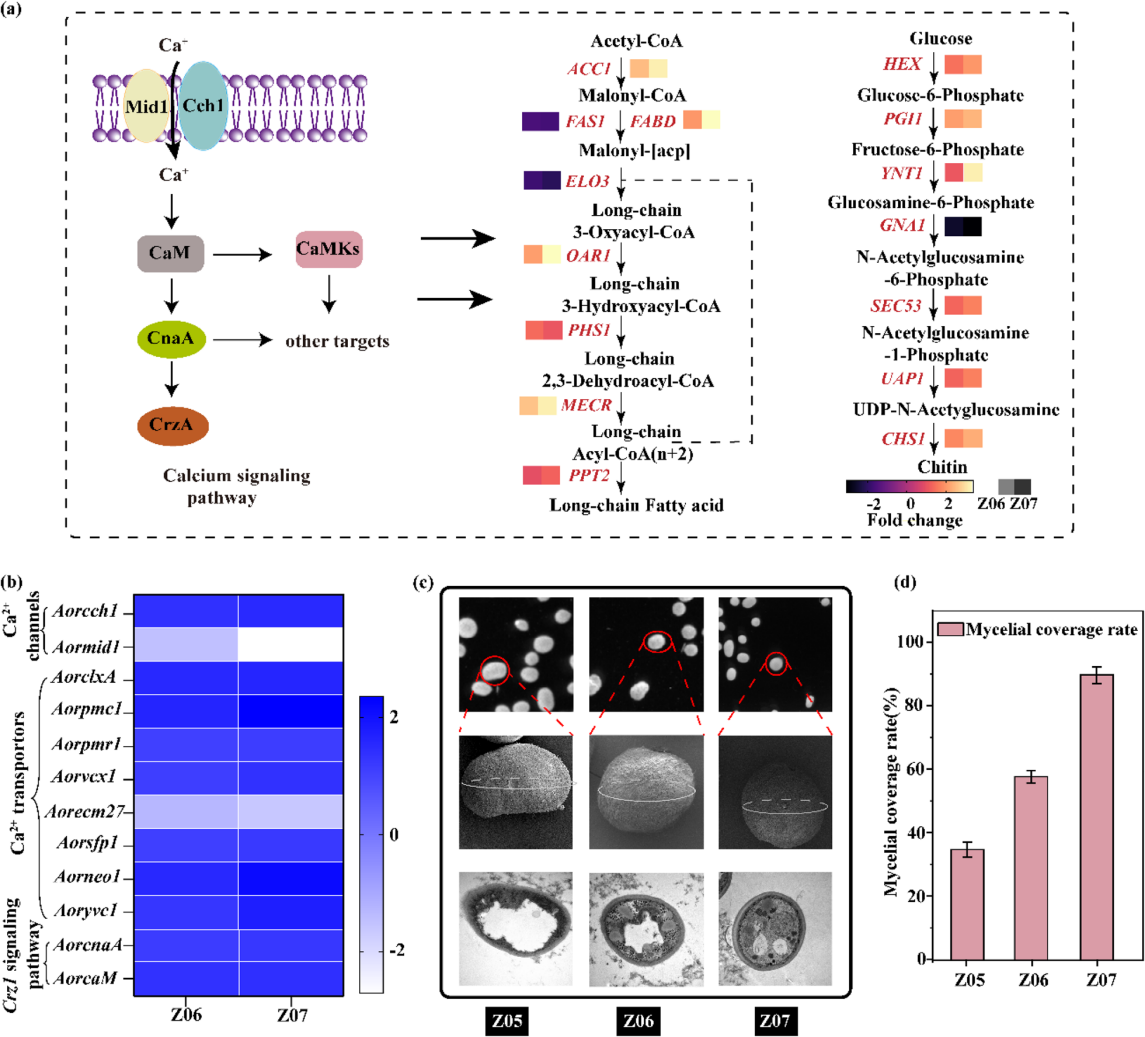
Transcriptome analysis and morphological characterization to clarify the mechanism of mycelial pellets formation in *Aspergillus oryzae*.** a** Diagram of the mechanism of mycelial pellets. **b** Heat map of the mycelial pellets formation mechanism following comparison between strain Z06 and Z07. **c** The morphological characteristics of mycelial pellets of the strain Z05, Z06 and Z07 under scanning electron microscope. **d** Mycelial coverage rate of strain Z05, Z06 and Z07. All data are presented as mean values from three independent experiments. Error bars indicate the standard deviations

To elucidate the genes’ response to mycelial pellets, the transcriptome data of strains Z05, Z06, and Z07 were compared. The expression level of most genes related to the calcium signaling pathway was significantly increased (Fig. 6b), including genes related to the Ca^2+^ transporter, such as *Aoryvc1* (Z06: 1.28-fold, Z07: 1.75-fold), *AorclxA* (Z06: 1.55-fold, Z07: 1.62-fold), *Aorpcm1* (Z06: 1.63-fold, Z07: 2.36-fold), *Aorvcx1* (Z06: 1.12-fold, Z07: 1.37-fold), and *Aorpmr1* (Z06: 1.09-fold, Z07: 1.14-fold), and genes related to regulating *CrzA*, such as *AorcnaA* (Z06: 1.16-fold, Z07: 1.25-fold) and *AorcaM* (Z06: 1.37-fold, Z07: 1.42-fold). The expression level of *Aorpcm1* was upregulated 1.63-fold and 2.36-fold in strains Z06 and Z07, respectively. Furthermore, the expression level of the genes for lipids synthesis, such as *Aoracc1* (Z06: 2.63-fold, Z07: 3.31-fold), *AorfabD* (Z06: 2.17-fold, Z07: 3.52-fold), *Aoroar1* (Z06: 2.27-fold, Z07: 3.53-fold), *Aorphs1* (Z06: 1.18-fold, Z07: 1.51-fold), and *AormecR* (Z06: 2.77-fold, Z07: 3.38-fold), and genes for chitin synthesis, such as *Aorhex* (Z06: 1.67-fold, Z07: 2.12-fold), *Aorpgi1* (Z06: 2.26-fold, Z07: 2.59-fold), *Aorynt1* (Z06: 1.23-fold, Z07: 3.34-fold), *Aoruap1* (Z06: 1.46-fold, Z07: 1.87-fold), and *Aorchs1* (Z06: 1.91-fold, Z07: 2.46-fold), were increased in strains Z06 and Z07 (Fig. 6a).

The pellets were observed by SEM and their surface did not change significantly. When the pellets were cut to observe the internal mycelial coverage, the area covered by mycelia inside the pellets was gradually increased, and the pellets were more compact (Fig. 6c). The specific data also showed that mycelial coverage of the pellets in strains Z06 and Z07 reached 58.7–90.1%, respectively, which were 1.66 and 2.57 times higher than that of strain Z05 (Fig. 6d). The mycelia inside the pellets were essential for nutrient and oxygen uptake (Schmideder et al. 2021), indicating that the evolved strains Z06 and Z07 exhibited better mycelial pellet formation.

## Discussion

In this study, the optimal range of *A. oryzae* growth parameters for L-malate efficient production was determined first. Then, the mutant strain Z07 with the optimal growth parameters was obtained through adaptive evolution, with an L-malate titer, yield, and productivity of 132.5 g/L, 0.79 g/g, and 1.1 g/L/h, respectively. Further, transcriptome data and morphological characterization were used to elucidate the mechanisms of how the growth status affected the L-malate production. The results present a new insight into L-malate synthesis using *A. oryzae* and may provide a new strategy for precisely controlling filamentous fungus growth in industrial fermentation.

The relationship between colony diameter, PVM value, and pellet number of *A. oryzae* and L-malate production was determined in this study. Previous studies have shown that the growth status of filamentous fungi strongly affects the capacity to produce target compounds (Driouch et al. 2010; Teng et al. 2009). The growth phenotype of filamentous fungi is mainly divided into three levels (colony, pellets, and spore) and can be assessed based on six indicators, including mycelium development (Fiedler et al. 2018), pellet diameter (Zhou et al. 2011), pellet number (Papagianni 2004), pellet-specific surface area (Ding et al. 2018), spore color (Ottenheim et al. 2015), and surface smoothness (Chen et al. 2019). For example, the pellet diameter of *Rhizopus delemar* was the key factor for fumaric acid production, when the concentration of glucose and soybean peptone was optimized to 20 g/L and 6 g/L to optimize the diameter and biomass of the pellets, the fumaric acid titer and productivity increased to 35.42 g/L and 0.49 g/L/h, respectively (Zhou et al. 2011). Similarly, the pellet number of *Penicillium flavum* exhibited a significant effect on penicillin production, and an appropriate number of pellets (10^4^/mL) was achieved by changing the concentration of inoculated spores, then the penicillin titer was increased from 500 U/mL to 5000 U/mL (Papagianni 2004). In *A. niger*, mycelial development affects protein secretion. The protein secretion of *A. niger* was increased with changing the expression levels of the key gene *arfA* (Fiedler et al. 2018). These examples further demonstrate that the growth phenotype of filamentous fungi is key to efficiently synthesizing targeted chemicals.

The growth mechanism of hyphal branching, conidiophores, and mycelial pellet formation were elucidated in this study. First, the key MAPK signaling module Anfus3-AnSte7-AnSte11 with the adaptor protein AnSte50 was actively expressed in *A. oryzae*, which causes active expression of the intracellular DNA synthesis pathway and ultimately affects hyphal branching formation in *A. oryzae*. The main factors affecting hyphal branching in filamentous fungi are the MAPK signaling pathway (Bayram et al. 2012), light signals (Purschwitz et al. 2006), reactive oxygen species (Semighini and Harris 2008), and oxylipins (Tsitsigiannis and Keller 2007). The MAPK signaling pathway module in *A. niger* consists of the MAP kinase AnFus3, the upstream kinases AnSte7 and AnSte11, and the AnSte50 adapter and controls fungal development and coordinates to produce secondary metabolites in *A. niger* (Bayram et al. 2012)*.* Second, conidiophore formation in *A. oryzae* was affected by the central regulatory pathway *brlA* → *abaA* → *wetA*, the upstream regulators *fluG*, *flbA*, *flbB*, *flbC*, and *flbD*, and the negative regulators *sfgA*, *veA*, *velB*, *nsdC*, and *nsdD*. Literature mining showed that light regulation (Schmoll et al. 2010), the HogA signaling pathway (Vargas-Perez et al. 2007), the nitrogen metabolism pathway (Crescenzi et al. 1983), the conidiophore center regulation pathway (Han and Adams 2001; Ming-Yueh et al. 2018), and other regulators (Lee et al. 2014; Seo et al. 2003) in filamentous fungi control conidiophore formation. For example, the activation of *brlA*, which encodes a C_2_H_2_ zinc finger-type transcription factor in *Aspergillus* species, has been demonstrated to be a key signal for the induction of spore development formation (Han and Adams 2001). Third, the calcium signaling pathway was actively expressed in *A. oryzae*, which causes active expression of the cell wall and membrane formation and ultimately makes a certain impact on mycelial pellet formation in *A. oryzae*. Hydrophobic interactions (Dynesen and Nielsen 2003; Zhang and Zhang 2016), calcium signaling pathways (de Castro et al. 2014; Wang et al. 2012), and histone methylation (Palmer et al. 2008) control mycelial pellet formation in filamentous fungi. For example, when the hydrophobic proteins *rodA* and *dewA* in *A. nidulans* were knocked out, the diameter and biomass of mycelial pellets were reduced by 25% and 51%, respectively (Dynesen and Nielsen 2003). Similarly, CchA and MidA in *A. niger* contribute to the growth and significantly impact the regulation of pellet formation and cell wall synthesis (Wang et al. 2012). In addition, we speculated that changes in genes involved in growth mechanism may have an effect on genes related to the L-malate synthesis pathway, thus affecting the production of L-malate (Additional file 1: Fig. S1, S2).

## Conclusions

The growth status of *A. oryzae* was investigated to enhance the ability to produce L-malate. The correlation between the growth state of *A. oryzae* and L-malate production was analyzed, and then the optimal growth parameters for L-malate production were obtained about the range of 26–30 mm colony diameter, 35–40% PVM value and 220–240/ml pellets number. Adaptive evolution was used to improve the growth status of *A. oryzae* and the evolved strain Z07 was obtained, whose L-malate titer, L-malate yield, and L-malate productivity reached 132.5 g/L, 0.79 g/g and 1.1 g/L/h during 7.5 L fermentation. In addition, the growth mechanism of *A. oryzae* was further investigated by combining transcriptome analysis and morphological characterization. These results provide new insights into the potential for development of filamentous fungi for microbial production of other chemicals.

### Supplementary Information

**Additional file 1**: **Fig. S1.** The synthesis routes of L-malate. **Fig. S2.** Heat map of L-malate synthesis pathway following comparison between the strain Z06 and Z07.

## Data Availability

All data generated or analyzed during this study are included in this article.

## References

[CR1] Battat E, Peleg Y, Bercovitz A, Rokem JS, Goldberg I (1991). Optimizatiom of L-malic acid production by *Aspergillus-flavus* in a stirred fermenter. Biotechnol Bioeng.

[CR2] Bayram O, Bayram OS, Ahmed YL, Maruyama J, Valerius O, Rizzoli SO, Ficner R, Irniger S, Braus GH (2012). The *Aspergillus nidulans* MAPK module AnSte11-Ste50-Ste7-Fus3 controls development and secondary metabolism. PLoS Genet.

[CR3] Brown SH, Bashkirova L, Berka R, Chandler T, Doty T, McCall K, McCulloch M, McFarland S, Thompson S, Yaver D, Berry A (2013). Metabolic engineering of *Aspergillus oryzae* NRRL 3488 for increased production of L-malic acid. Appl Microbiol Biotechnol.

[CR4] Chen X, Zhou J, Ding Q, Luo Q, Liu L (2019). Morphology engineering of *Aspergillus oryzae* for L-malate production. Biotechnol Bioeng.

[CR5] Crescenzi O, Kurtz M, Champe S (1983). Developmental defects resulting from arginine auxotrophy in *Aspergillus nidulans*. J Gen Microbiol.

[CR6] de Castro PA, Chiaratto J, Winkelstroter LK, Bom VL, Ramalho LN, Goldman MH, Brown NA, Goldman GH (2014). The involvement of the Mid1/Cch1/Yvc1 calcium channels in *Aspergillus fumigatus* virulence. PLoS ONE.

[CR7] Ding Q, Luo Q, Zhou J, Chen X, Liu L (2018). Enhancing L-malate production of *Aspergillus oryzae* FMME218-37 by improving inorganic nitrogen utilization. Appl Microbiol Biotechnol.

[CR8] Dong X, Chen X, Qian Y, Wang Y, Wang L, Qiao W, Liu L (2017). Metabolic engineering of *Escherichia coli* W3110 to produce L-malate. Biotechnol Bioeng.

[CR9] Dörsam S, Fesseler J, Gorte O, Hahn T, Zibek S, Syldatk C, Ochsenreither K (2017). Sustainable carbon sources for microbial organic acid production with filamentous fungi. Biotechnol Biofuels.

[CR10] Driouch H, Sommer B, Wittmann C (2010). Morphology engineering of *Aspergillus niger* for improved enzyme production. Biotechnol Bioeng.

[CR11] Dynesen J, Nielsen J (2003). Surface hydrophobicity of *Aspergillus nidulans* conidiospores and its role in pellet formation. Biotechnol Prog.

[CR12] Fiedler MRM, Cairns TC, Koch O, Kubisch C, Meyer V (2018). Conditional expression of the small GTPase ArfA impacts secretion, morphology, growth, and actin ring position in *Aspergillus niger*. Front Microbiol.

[CR13] Goldberg I, Rokem JS, Pines O (2006). Organic acids: old metabolites, new themes. J Chem Technol Biot.

[CR14] Guo L, Zhang F, Zhang C, Hu G, Gao C, Chen X, Liu L (2018). Enhancement of malate production through engineering of the periplasmic rTCA pathway in *Escherichia coli*. Biotechnol Bioeng.

[CR15] Han S, Adams TH (2001). Complex control of the developmental regulatory locus *brlA* in *Aspergillus nidulans*. Mol Genet Genomics.

[CR16] Iyyappan J, Bharathiraja B, Baskar G, Jayamuthunagai J, Barathkumar S, Anna Shiny R (2018). Malic acid production by chemically induced *Aspergillus niger* MTCC 281 mutant from crude glycerol. Bioresour Technol.

[CR17] Ji L, Wang J, Luo Q, Ding Q, Tang W, Chen X, Liu L (2021). Enhancing L-malate production of *Aspergillus oryzae* by nitrogen regulation strategy. Appl Microbiol Biotechnol.

[CR18] Knuf C, Nookaew I, Brown SH, McCulloch M, Berry A, Nielsen J (2013). Investigation of malic acid production in *Aspergillus oryzae* under nitrogen starvation conditions. Appl Environ Microbiol.

[CR19] Lee MK, Kwon NJ, Choi JM, Lee IS, Jung S, Yu JH (2014). NsdD is a key repressor of asexual development in *Aspergillus nidulans*. Genetics.

[CR20] Liu J, Xie Z, Shin HD, Li J, Du G, Chen J, Liu L (2017). Rewiring the reductive tricarboxylic acid pathway and L-malate transport pathway of *Aspergillus oryzae* for overproduction of L-malate. J Biotechnol.

[CR21] Liu J, Li J, Liu Y, Shin HD, Ledesma-Amaro R, Du G, Chen J, Liu L (2018). Synergistic rewiring of carbon metabolism and redox metabolism in cytoplasm and mitochondria of *Aspergillus oryzae* for increased L-malate production. ACS Synth Biol.

[CR22] Ljubimova JY, Fujita M, Khazenzon NM, Lee BS, Wachsmann-Hogiu S, Farkas DL, Black KL, Holler E (2008). Nanoconjugate based on polymalic acid for tumor targeting. Chem Biol Interact.

[CR23] Ming-Yueh W, Matthew E, Mi-Kyung L, Erin M, Sun-Chang K, Jae-Hyuk Y (2018). Systematic dissection of the evolutionarily conserved WetA developmental regulator across a genus of filamentous fungi. mBio.

[CR24] Nakayama S, Tabata K, Oba T, Kusumoto K, Mitsuiki S, Kadokura T, Nakazato A (2012). Characteristics of the high malic acid production mechanism in *Saccharomyces cerevisiae* sake yeast strain No. 28. J Biosci Bioeng.

[CR25] Ochsenreither K, Fischer C, Neumann A, Syldatk C (2014). Process characterization and influence of alternative carbon sources and carbon-to-nitrogen ratio on organic acid production by *Aspergillus oryzae* DSM1863. Appl Microbiol Biotechnol.

[CR26] Ottenheim C, Werner K, Zimmermann W, Wu J (2015). Improved endoxylanase production and colony morphology of *Aspergillus niger* DSM 26641 by γ-ray induced mutagenesis. Biochem Eng J.

[CR27] Palmer JM, Perrin RM, Dagenais TR, Keller NP (2008). H3K9 methylation regulates growth and development in *Aspergillus fumigatus*. Eukaryot Cell.

[CR28] Papagianni M (2004). Fungal morphology and metabolite production in submerged mycelial processes. Biotechnol Adv.

[CR29] Pringle A, Taylor J (2002). The fitness of filamentous fungi. Trends Microbiol.

[CR30] Purschwitz J, Muller S, Kastner C, Fischer R (2006). Seeing the rainbow: light sensing in fungi. Curr Opin Microbiol.

[CR31] Schmideder S, Muller H, Barthel L, Friedrich T, Niessen L, Meyer V, Briesen H (2021). Universal law for diffusive mass transport through mycelial networks. Biotechnol Bioeng.

[CR32] Schmoll M, Esquivel-Naranjo EU, Herrera-Estrella A (2010). Trichoderma in the light of day–physiology and development. Fungal Genet Biol.

[CR33] Schoustra S, Punzalan D (2012). Correlation of mycelial growth rate with other phenotypic characters in evolved genotypes of *Aspergillus nidulans*. Fungal Biol.

[CR34] Semighini CP, Harris SD (2008). Regulation of apical dominance in *Aspergillus nidulans* hyphae by reactive oxygen species. Genetics.

[CR35] Seo JA, Guan Y, Yu JH (2003). Suppressor mutations bypass the requirement of fluG for asexual sporulation and sterigmatocystin production in *Aspergillus nidulans*. Genetics.

[CR36] Stark D, Zala D, Münch T, Sonnleitner B, Marison I, Stockar U (2003). Inhibition aspects of the bioconversion of l-phenylalanine to 2-phenylethanol by *Saccharomyces cerevisiae*. Enzyme Microb Technol.

[CR37] Taing O, Taing K (2006). Production of malic and succinic acids by sugar-tolerant yeast *Zygosaccharomyces rouxii*. Eur Food Res Technol.

[CR38] Teng Y, Xu Y, Wang D (2009). Changes in morphology of *Rhizopus chinensis* in submerged fermentation and their effect on production of mycelium-bound lipase. Bioprocess Biosyst Eng.

[CR39] Thakker C, Martinez I, Li W, San KY, Bennett GN (2015). Metabolic engineering of carbon and redox flow in the production of small organic acids. J Ind Microbiol Biotechnol.

[CR40] Tsitsigiannis DI, Keller NP (2007). Oxylipins as developmental and host-fungal communication signals. Trends Microbiol.

[CR41] Vargas-Perez I, Sanchez O, Kawasaki L, Georgellis D, Aguirre J (2007). Response regulators SrrA and SskA are central components of a phosphorelay system involved in stress signal transduction and asexual sporulation in *Aspergillus nidulans*. Eukaryot Cell.

[CR42] Wang S, Cao J, Liu X, Hu H, Shi J, Zhang S, Keller NP, Lu L (2012). Putative calcium channels CchA and MidA play the important roles in conidiation, hyphal polarity and cell wall components in *Aspergillus nidulans*. PLoS ONE.

[CR43] West TP (2011). Malic acid production from thin stillage by *Aspergillus* species. Biotechnol Lett.

[CR44] Xu Y, Zhou Y, Cao W, Liu H (2020). Improved production of malic acid in *Aspergillus niger* by abolishing citric acid accumulation and enhancing glycolytic flux. ACS Synth Biol.

[CR45] Ye X, Honda K, Morimoto Y, Okano K, Ohtake H (2013). Direct conversion of glucose to malate by synthetic metabolic engineering. J Biotechnol.

[CR46] Zambanini T, Sarikaya E, Kleineberg W, Buescher JM, Meurer G, Wierckx N, Blank LM (2016). Efficient malic acid production from glycerol with *Ustilago trichophora* TZ1. Biotechnol Biofuels.

[CR47] Zambanini T, Hosseinpour Tehrani H, Geiser E, Sonntag CK, Buescher JM, Meurer G, Wierckx N, Blank LM (2017). Metabolic engineering of *Ustilago trichophora* TZ1 for improved malic acid production. Metab Eng Commun.

[CR48] Zhang J, Zhang J (2016). The filamentous fungal pellet and forces driving its formation. Crit Rev Biotechnol.

[CR49] Zhang X, Wang X, Shanmugam KT, Ingram LO (2011). L-malate production by metabolically engineered *Escherichia coli*. Appl Environ Microbiol.

[CR50] Zhou Z, Du G, Hua Z, Zhou J, Chen J (2011). Optimization of fumaric acid production by *Rhizopus delemar* based on the morphology formation. Bioresour Technol.

