# Supplementary material for: Engineering growth phenotypes of Aspergillus oryzae for L-malate production
Source: Bioresour Bioprocess. 2023 Apr 5;10(1):25. doi: 10.1186/s40643-023-00642-7 (PMC10991988; doi:10.1186/s40643-023-00642-7)
Supplement: Supplementary file 1 — Additional file 1: Fig. S1. The synthesis routes of L-malate. Fig. S2. Heat map of L-malate synthesis pathway following comparison between the strain Z06 and Z07. [file 40643_2023_642_MOESM1_ESM.docx]

**Engineering** **growth phenotypes of *Aspergillus oryzae* for L-malate production**

Huiyun Zuo^1,2^, Lihao Ji^1,2^, Jingyu Pan^1,2^, Xiulai Chen^1,2^, Cong Gao^1,2^, Liang Guo^1,2^, Jia Liu^1,2^, Wanqing Wei^1,2^, Jing Wu^3^, Wei Song^3^, Liming Liu^1,2*^.

^1^State Key Laboratory of Food Science and Technology, Jiangnan University, Wuxi, Jiangsu 214122, China

^2^ International Joint Laboratory on Food Safety, Jiangnan University, Wuxi 214122, China

^3^School of Pharmaceutical Science, Jiangnan University, Wuxi, Jiangsu 214122, China

*** Corresponding author.**

State Key Laboratory of Food Science and Technology, Jiangnan University, 1800 Lihu Road, Wuxi 214122, China.

Fax/Tel.: +86-510-85197875.

E-mail: mingll@jiangnan.edu.cn (Liming Liu).

This doc file includes:

Supplementary Figure 1 to Figure 2

# Supplementary Figures


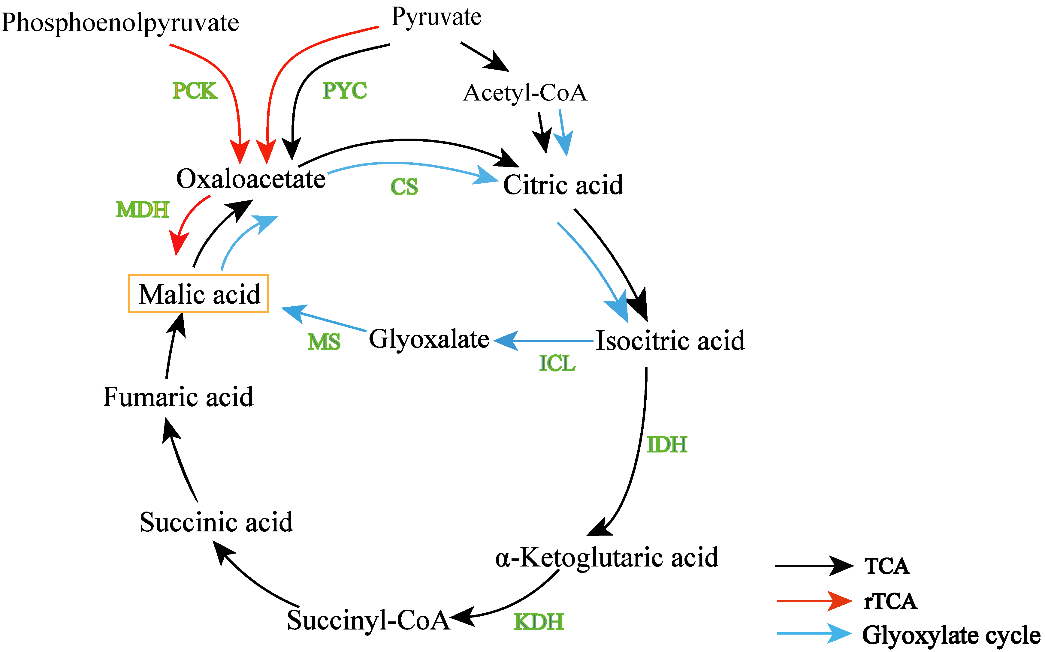


**Fig. S1.** The synthesis routes of L-malate.


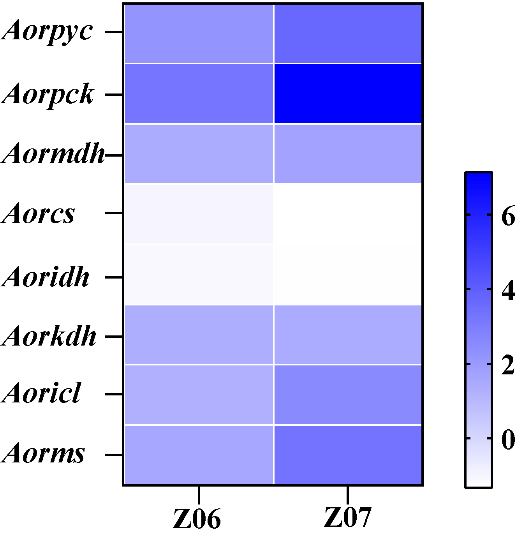


**Fig. S2.** Heat map of L-malate synthesis pathway following comparison between the strain Z06 and Z07
